# Preparation and X-ray structure of 2-iodoxybenzenesulfonic acid (IBS) – a powerful hypervalent iodine(V) oxidant

**DOI:** 10.3762/bjoc.14.159

**Published:** 2018-07-20

**Authors:** Irina A Mironova, Pavel S Postnikov, Rosa Y Yusubova, Akira Yoshimura, Thomas Wirth, Viktor V Zhdankin, Victor N Nemykin, Mekhman S Yusubov

**Affiliations:** 1 The Tomsk Polytechnic University, 634050 Tomsk, Russia; 2 School of Chemistry, Cardiff University Park Place, Main Building, Cardiff CF10 3AT, UK; 3 Department of Chemistry and Biochemistry, University of Minnesota Duluth, MN 55812, USA; 4 Department of Chemistry, University of Manitoba, Winnipeg, MB R3T 2N2, Canada

**Keywords:** hypervalent iodine, iodine, 2-iodoxybenzenesulfonic acid, oxidation, X-ray

## Abstract

The selective preparation of 2-iodoxybenzenesulfonic acid (IBS, as potassium or sodium salts) by oxidation of sodium 2-iodobenzenesulfonate with Oxone or sodium periodate in water is reported. The single crystal X-ray diffraction analysis reveals a complex polymeric structure consisting of three units of IBS as potassium salt and one unit of 2-iodoxybenzenesulfonic acid linked together by relatively strong I=O···I intermolecular interactions. Furthermore, a new method for the preparation of the reduced form of IBS, 2-iodosylbenzenesulfonic acid, by using periodic acid as an oxidant, has been developed. It has been demonstrated that the oxidation of free 2-iodobenzenesulfonic acid under acidic conditions affords an iodine(III) heterocycle (2-iodosylbenzenesulfonic acid), while the oxidation of sodium 2-iodobenzenesulfonate in neutral aqueous solution gives the iodine(V) products.

## Introduction

Recently, the interest in synthetic applications of hypervalent iodine compounds as stoichiometric reagents or catalysts has experienced an explosive growth [1–8]. Hypervalent iodine(V) compounds represent an important class of oxidative reagents extensively employed in organic synthesis [9–11]. 2-Iodoxybenzoic acid (IBX) and the product of its acetylation Dess–Martin periodinane (DMP) are the most common oxidants used for selective oxidation of alcohols to carbonyl compounds as well as for a variety of other synthetically useful oxidative transformations [10–11]. IBX and DMP are mild oxidants with a relatively low reactivity towards some substrates. Moreover, these reagents are generally not suitable as active species in catalytic reactions due to the low reactivity and harsh conditions required for their in situ generation. In 2009, Ishihara and co-workers have reported an extremely active catalytic system for oxidation of alcohols based on 2-iodoxybenzenesulfonic acid (IBS) as the active species [12–13]. IBS (or its sodium salt) is much more active as catalyst than IBX derivatives. In particular, it can be used as a highly efficient and selective catalyst (0.05–5 mol %) for the oxidation of primary and secondary alcohols to the respective carbonyl compounds with Oxone^®^ (2KHSO_5_·KHSO_4_·K_2_SO_4_) in nitromethane, acetonitrile, or ethyl acetate [13]. Recent research has revealed the extreme activity of IBS as a catalyst in numerous other oxidations, such as: the oxidation of benzylic and alkane C–H bonds [14], the oxidation of phenols to 1,2-quinones [15], the cyclization and cross-coupling reactions [16], and the site-selective hydroxylative dearomatization of 2-substituted phenols to either 1,2-benzoquinols or their cyclodimers [17].

**Scheme 1:**
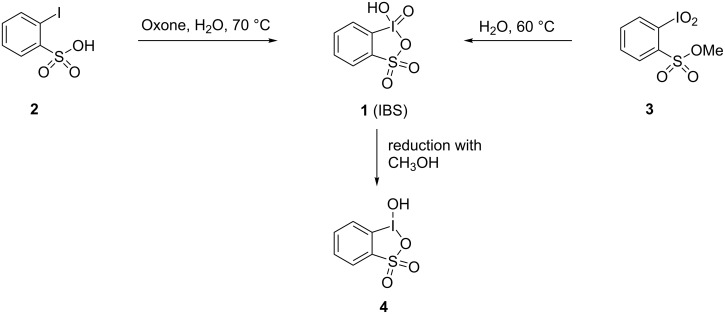
Previously reported preparation of IBS (**1**) [17].

The first preparation and isolation of IBS (**1**) was attempted in 2006 using two different approaches: direct oxidation of 2-iodobenzenesulfonic acid (**2**) by Oxone or hydrolysis of methyl 2-iodoxybenzenesulfate (**3**, Scheme 1) [18].

The hydrolysis of sulfonic ester **3** forms IBS as a mixture with methanol which is quickly oxidized by IBS in situ producing the corresponding iodine(III) heterocycle, 2-iodosylbenzenesulfonic acid (**4**) as main product. The direct oxidation of 2-iodobenzenesulfonic acid (**2**) with Oxone leads to the formation of the desired IBS (**1**, Scheme 1), however, contaminated with inorganic impurities. Because of the high solubility of IBS in water, this mixture is difficult to separate. The resulting IBS is insoluble in nonpolar solvents (dichloromethane, chloroform, etc.). Moreover, IBS has high reactivity towards polar organic solvents (acetonitrile, DMSO, methanol) being readily reduced to 2-iodosylbenzenesulfonic acid upon contact with these solvents. Despite these problems, IBS was previously characterized by ^1^H and ^13^C NMR, IR spectroscopy, high-resolution mass spectrometry, and elemental analysis. However, all previously reported attempts to grow single crystals of IBS from methanol or acetonitrile resulted in reduction with the formation of 2-iodosylbenzenesulfonic acid as confirmed by X-ray diffraction analysis [17]. In the present work, we report the preparation and isolation of IBS (as potassium or sodium salts) and its structural study by X-ray analysis. Furthermore, we have developed a new method for the preparation of the IBS reduced form, 2-iodosylbenzenesulfonic acid (**4**), with the use of periodic acid as an oxidant.

## Results and Discussion

**Scheme 2:**
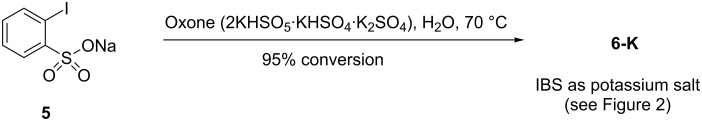
Oxidation of 2-iodobenzenesulfonate **5** by Oxone in water.

We have investigated the oxidation of 2-iodobenzenesulfonic acid as sodium salt and as a free acid using Oxone, sodium periodate or periodic acid. The oxidation of sodium 2-iodobenzenesulfonate (**5**) by Oxone was performed under Ishihara's conditions [13] in water at 70 °C (Scheme 2). NMR monitoring indicated 95% conversion of the starting sodium salt **5** to the iodine(V) product **6** after about 3 h stirring at 70 °C (Figure S1 in Supporting Information File 1). After cooling the aqueous solution to room temperature, the formation of a precipitate consisting of needle-shaped organic crystals and microcrystalline powder of inorganic salts was observed. The needle-shaped organic crystals were manually separated from the inorganic salts and analysed by NMR spectroscopy and X-ray crystallography. ^1^H and ^13^C NMR spectra of these crystals were in full agreement with the NMR spectra of IBS provided in the Supporting Information of Ishihara's paper [13] and, in particular, displayed the characteristic signals of the *ortho-*protons (relative to iodine(V)) at 8.28 ppm.

**Figure 1:**
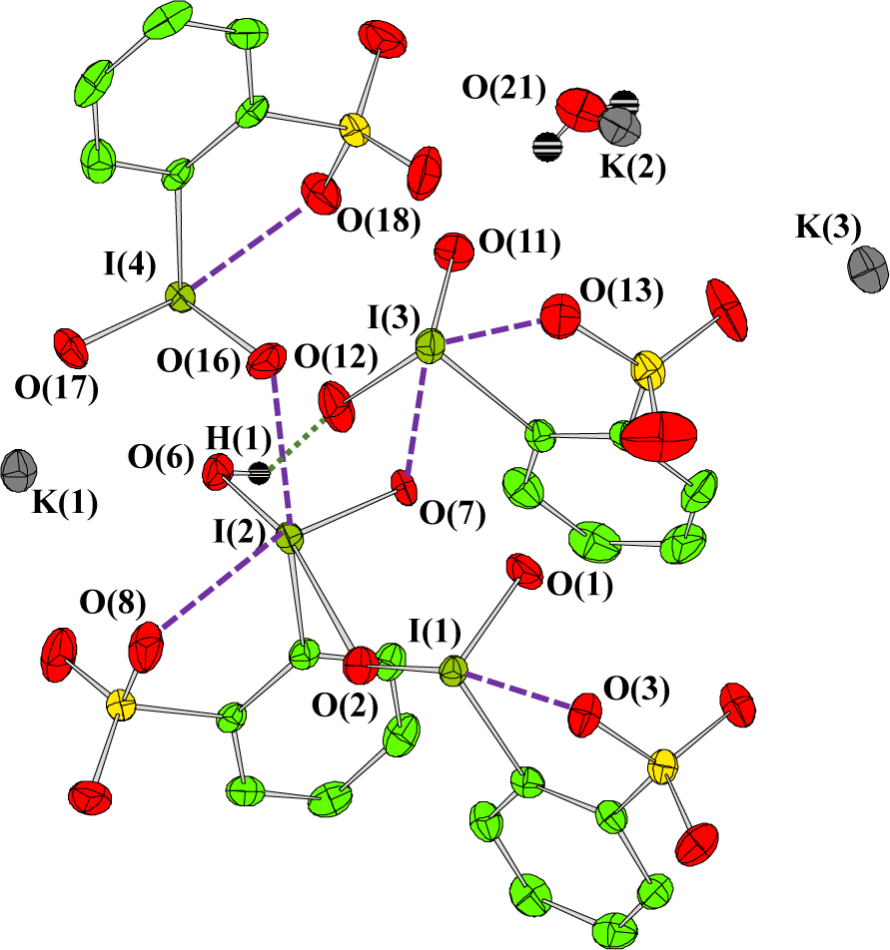
X-ray structure of an independent crystal unit of IBS **6-K**.

**Figure 2:**
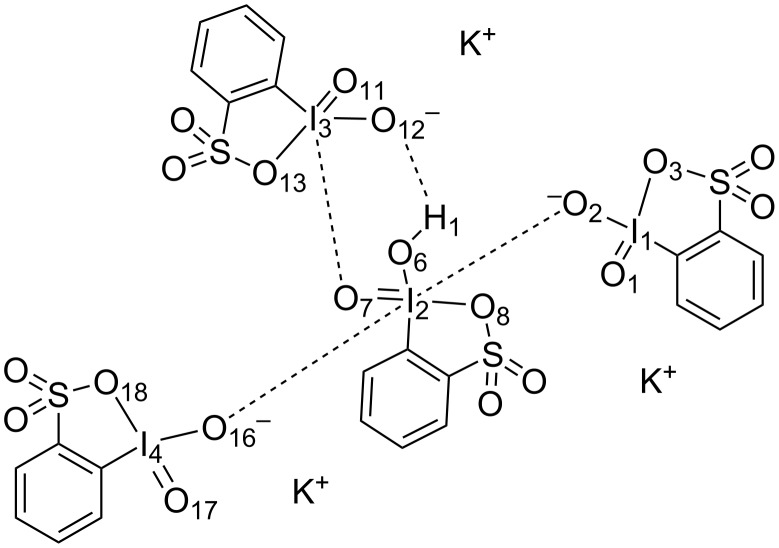
Simplified representation of structure **6-K**. Selected interatomic distances (Å): I(1)=O(1) 1.79; I(1)=O(2) 1.81; I(1)–O(3) 2.54; O(2)–I(2) 2.46; I(2)=O(7) 1.81; I(2)–O(6) 1.88; I(2)–O(8) 2.60; I(2)–O(16) 2.71; I(3)=O(11) 1.80; I(3)=O(12) 1.87; I(3)–O(13) 2.49; I(3)–O(7) 2.63; I(4)=O(17) 1.8; I(4)=O(16) 1.81; I(4)–O(18) 2.60.

The structure of IBS-K crystals obtained by this reaction (Scheme 2) was established by single-crystal X-ray crystallography (for crystallographic details, see Table S1 in Supporting Information File 1). The X-ray structure of an independent unit of IBS-K is shown in Figure 1, and the corresponding structural drawing with interatomic bond distances is presented in Figure 2.

The X-ray crystal structure **6-K** is quite interesting. Each independent unit consists of a tetrameric aggregate, which holds together by relatively strong I=O···I intermolecular interactions formed between molecules of **6** (Figure 2). Two out of three molecules of **6** (those that include I(1) and I(4) centers) have very similar structures that consist of tetracoordinated iodine centers with two short (≈1.8 Å) I=O bonds, one regular I–C bond and one relatively long (≈2.54–2.60 Å) I···OSO_2_ interaction. In each fragment, one of the I=O oxygen atoms (O(2) and O(16)) forms a long (≈2.46 Å for I(1)=O(2)···I(2) and ≈2.71 Å for I(4)=O(16)···I(2)) secondary contact with the I(2) center. In contrast, the third molecule of **6** has pentacoordinated iodine(V) center formed by two short I(3)=O bonds, one I–C bond, a relatively long (≈2.49 Å) I···OSO_2_ intramolecular interaction and a relatively long (≈2.63 Å) I(2)=O(7)···I(3) intermolecular interactions. Unlike I(1)=O and I(4)=O centers, the I(3)–O(12) bond distance is significantly longer (≈1.87 Å) compared to the other I=O bond distances (≈1.80 Å), which is explained below. Finally, the “inner” I(2) center is hexacoordinated with two short (≈1.81 Å for I(2)=O(7) and ≈1.88 Å for I(2)–O(6)) bonds, one regular I(2)–C bond, one usual (2.60 Å) I(2)···OSO_2_ intramolecular contact and two (≈2.71 Å for I(4)=O(16)···I(2) and ≈2.46 Å for I(1)=O(2)···I(2)) intermolecular contacts. Three potassium ions were also identified in the crystal structure of **6**. In addition, a water molecule was observed in the crystal structure of **6**, which forms two strong hydrogen bonds (≈2.05 Å for O(21)–H(2)···O(11) and ≈2.07 Å for O(21)–H(3)···O(5)) with neighbouring oxygen atoms and two short donor–acceptor interactions with K(1) and K(2) potassium ions. Overall, the structure of the aggregate is indicative of only three potassium counterions and a neutral water molecule thus leaving the anionic iodine-containing tetramer. Careful examination of the electron density map and the geometry of this tetramer indicates on the two rather long I(2)–O(6) and I(3)–O(12) bonds (≈1.87–1.88 Å), their short (≈2.59 Å) O(6)···O(12) distance, and a small electron density close to O(6) and O(12). Thus, a proton was added at the small electron density region to the O(6) atom. Such proton positioning results in the formation of an expected strong hydrogen bond (≈1.87 Å ) in the O(6)–H(1)···O(12) fragment.

Manual separation of organic crystals of **6-K** from inorganic salts resulting from reduction of Oxone is a time-consuming, impractical procedure. Therefore, we investigated the use of oxidants different from Oxone for the oxidation of sodium 2-iodobenzenesulfonate (**5**). It is known from the literature that sodium periodate can oxidize various ArI to ArIO_2_ in boiling water or aqueous acetic acid [19–20]. We have found that according to NMR monitoring (Figure S2 in Supporting Information File 1), the reaction of sodium 2-iodobenzenesulfonate (**5**) with sodium periodate in water selectively affords the respective iodine(V) product after heating at 60 °C for 16 h with almost quantitative conversion. The aqueous solution containing IBS-Na (as sodium salt) and inorganic products resulting from the reduction of NaIO_4_ was treated with silver nitrate to precipitate I^–^ and IO_3_^–^ anions. The precipitate of silver salts was filtered off, and the mother liquor was concentrated using blowing air to about half of the initial volume. The concentrated aqueous solution was left for several days resulting in the formation of a microcrystalline precipitate of IBS-Na isolated in 61% yield. ^1^H and ^13^C NMR spectra of this product in D_2_O were identical to the spectra of IBS-K (**6-K**). Oxidation of sodium 2-iodobenzenesulfonate **5** with periodic acid under similar conditions afforded **6-Na** in a mixture with 2-iodosylbenzenesulfonic acid (**4**, see Figure S3 in Supporting Information File 1).

In order to avoid the formation of salts, we have investigated the oxidation of free 2-iodobenzenesulfonic acid with periodic acid (H_5_IO_6_). 2-Iodobenzenesulfonic acid (**7**) was prepared from sodium 2-iodobenzenesulfonate (**5**) by ion exchange using Amberlyst 15 (Н^+^). The oxidation reaction was carried out at 60 °С and monitored by NMR (Figure S5 in Supporting Information File 1). To our surprise, only the organoiodine(III) product 2-iodosylbenzenesulfonic acid (**4**) was formed under these conditions. Product **4** precipitated from the reaction mixture upon cooling to room temperature and was isolated in 87% yield by simple filtration. ^1^H and ^13^C NMR spectra of product **4** are identical to the previously reported spectroscopic data for 2-iodosylbenzenesulfonic acid [18,21–22]. In particular, the ^1^H NMR displayed the characteristic signal of the *ortho-*proton (relative to iodine(III)) at about 8.0 ppm and ^13^C NMR exhibited the *ipso* C–I(III) carbon at 112.1 ppm.

**Scheme 3:**
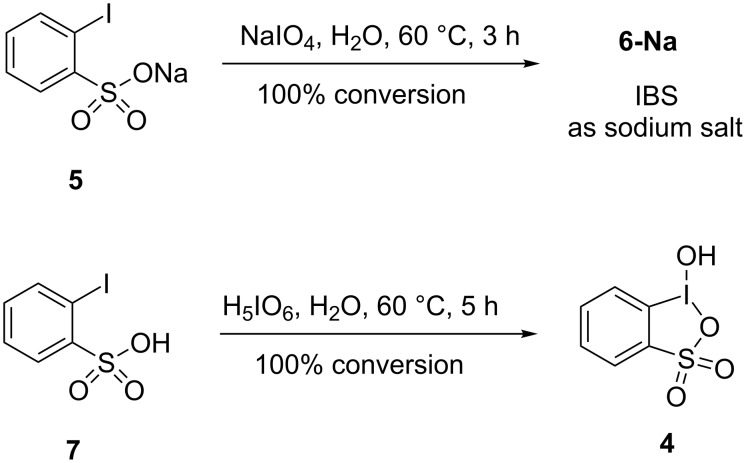
Comparison of the oxidation of sodium 2-iodobenzenesulfonate (**5**) with NaIO_4_ and 2-iodobenzenesulfonic acid (**7**) with H_5_IO_6_ in water.

These results clearly indicate that the oxidation of free 2-iodobenzenesulfonic acid affords iodine(III), while the oxidation of its salt gives the iodine(V) products (Scheme 3). It is a potentially important observation allowing to selectively synthesize IBS (as a salt) in neutral aqueous solution or 2-iodosylbenzenesulfonic acid under acidic conditions. This result can be explained by a greater stability of the heterocyclic molecule of 2-iodosylbenzenesulfonic acid towards disproportionation in comparison to the unknown salt of 2-iodosylbenzenesulfonic acid, which probably has a noncyclic (or pseudocyclic) structure of 2-OIC_6_H_4_SO_2_ONa. Indeed, X-ray analysis of 2-iodosylbenzenesulfonic acid (**4**) indicated a cyclic structure with a I–O bond length of 2.38 Å in the benziodoxathiole ring [18], while in the anionic iodine(V) benziodoxathiole ring in aggregate **6** (Figure 2) the I–O bonds are much longer (≈2.5–2.6 Å). It is known from the literature that the preparation of iodine(V) species from ArI and an oxidant involves initial formation of ArIO followed by disproportionation to ArI and ArIO_2_ [23]. Such disproportionation is significantly impeded or even impossible in the cyclic structure of 2-iodosylbenzenesulfonic acid.

## Conclusion

In conclusion, we have reported a selective preparation of IBS (as potassium or sodium salts) and investigated its structure by X-ray analysis. Furthermore, we have developed a new method for the preparation of the reduced form of IBS, 2-iodosylbenzenesulfonic acid, by using periodic acid as an oxidant. We have demonstrated that the oxidation of free 2-iodobenzenesulfonic acid under acidic conditions affords an iodine(III) heterocycle, while the oxidation of sodium 2-iodobenzenesulfonate in neutral aqueous solution gives the iodine(V) products.

## Supporting Information

File 1 Experimental details and NMR spectra.

File 2 Crystallographic information file of compound **6**.
