# Supplementary material for: Preparation and X-ray structure of 2-iodoxybenzenesulfonic acid (IBS) – a powerful hypervalent iodine(V) oxidant
Source: Beilstein J Org Chem. 2018 Jul 20;14:1854–8. doi: 10.3762/bjoc.14.159 (PMC6071709; doi:10.3762/bjoc.14.159)

Supporting Information

for

Preparation and X-ray structure of 2-iodoxybenzenesulfonic acid (IBS) – a powerful hypervalent iodine(V) oxidant

Irina A. Mironova[1], Pavel S. Postnikov[1], Rosa Y. Yusubova[1], Akira Yoshimura[1], Thomas Wirth[3], Viktor V. Zhdankin[1,2]*, Victor N. Nemykin[4] and Mekhman S. Yusubov[1]*

Address: [1]The Tomsk Polytechnic University, 634050 Tomsk, Russia; [2]Department of Chemistry and Biochemistry, University of Minnesota Duluth, MN 55812, USA; [3]School of Chemistry, Cardiff University Park Place, Main Building, Cardiff CF10 3AT, UK and [4]Department of Chemistry, University of Manitoba, Winnipeg, MB R3T 2N2, Canada

Email: Viktor V. Zhdankin - vzhdanki@d.umn.edu, Mekhman S. Yusubov* - yusubov@tpu.ru

* Corresponding author

Experimental details and NMR spectra

Table of Contents: **pp**

1. General experimental remarks **S2**

2. Preparation of IBS-K by Oxone oxidation **S2**

3. Preparation of IBS-Na by periodate oxidation **S6**

4. Preparation of 2-iodosylbenzenesulfonic acid **S9**

5. References **S10**

General experimental remarks

All reagents and solvents were from commercial sources and used without further purification from freshly opened containers. NMR spectra were recorded at 300 and 400 MHz (^{1}H NMR) and 75, 100 MHz (^{13}C NMR). Chemical shifts (δ) are reported in parts per million. Melting points were determined using a Büchi M-560 apparatus.

Preparation of sodium 2-iodobenzenesulfonate (5).[1] 2-Aminobenzenesulfonic acid (2 g, 11.6 mmol) was added to a solution of *p*-toluenesulfonic acid (6.59 g, 34.7 mmol) in 20 mL of acetonitrile, and the reaction mixture was stirred for 10 min. Then, the resulting suspension was cooled on an ice-bath to 10–15 °C and a solution of sodium nitrite (2.39 g, 34.7 mmol) and potassium iodide (4.82 g, 29 mmol) in 6 mL of distilled water was added gradually. The reaction mixture was stirred for 10 min, then, allowed to warm to 20 °C and stirred for an additional 1 hour. Then, sodium thiosulfate was added until the reaction mixture became colorless and sodium bicarbonate solution (1 M solution) was added until pH 9–10. The formed precipitate was filtered off, washed with hot ethanol (20 mL) and acetone (30 mL). Product **5** was obtained as an off-white solid; yield 3.212 g (91%); mp >350 °C. ^{1}H NMR (400 MHz, D_2O) δ, ppm: 8.06 (d, J = 7.6 Hz, 1H), 7.95 (d, J = 7.6 Hz, 1H), 7.45 (t, J = 7.6 Hz, 1H), 7.16 (t, J = 7.6 Hz, 1H). ^{13}C NMR (100 MHz, D_2O) δ, ppm: 144.8, 142.1, 132.4, 128.4, 128.3, 90.9.

Oxidation of sodium 2-iodobenzenesulfonate (5) by Oxone.

A solution of sodium 2-iodobenzenesulfonate (**5**, 116 mg, 0.38 mmol) was dissolved in 1.0 mL of D_2O and the Oxone (350 mg, 3 equiv) was added. The reaction mixture was stirred for 6 hours at 70 °C and monitored by ^{1}H NMR. After cooling the aqueous solution to room temperature, the formation of a precipitate consisting of needle-shaped organic crystals and microcrystalline powder of inorganic salts was observed. The needle-shaped organic crystals were manually separated from the inorganic salts and analysed by NMR spectroscopy and X-ray crystallography. Product **6-K** was obtained as an off-white solid; yield 86 mg (66%); mp 279-360 °C (dec). ^{1}H NMR (300 MHz, D_2O) δ, ppm: 8.24 (d, J =

8.1 Hz, 1H), 8.03-7.99 (m, 2H), 7.90 (t, *J* = 7.5 Hz, 1H). ^{13}C NMR (75 MHz, D_2O) δ, ppm: 142.7, 139.3, 134.2, 133.5, 128.2, 122.4. Literature data[2]: ^{1}H NMR (500 MHz, D_2O) δ, ppm: 8.29 (dd, 1 H), 8.05 (m, 2 H), 7.95 (m, 1 H). ^{13}C NMR (125 MHz, D_2O) δ, ppm: 143.1, 139.7, 134.5, 133.7, 128.5, 122.6.

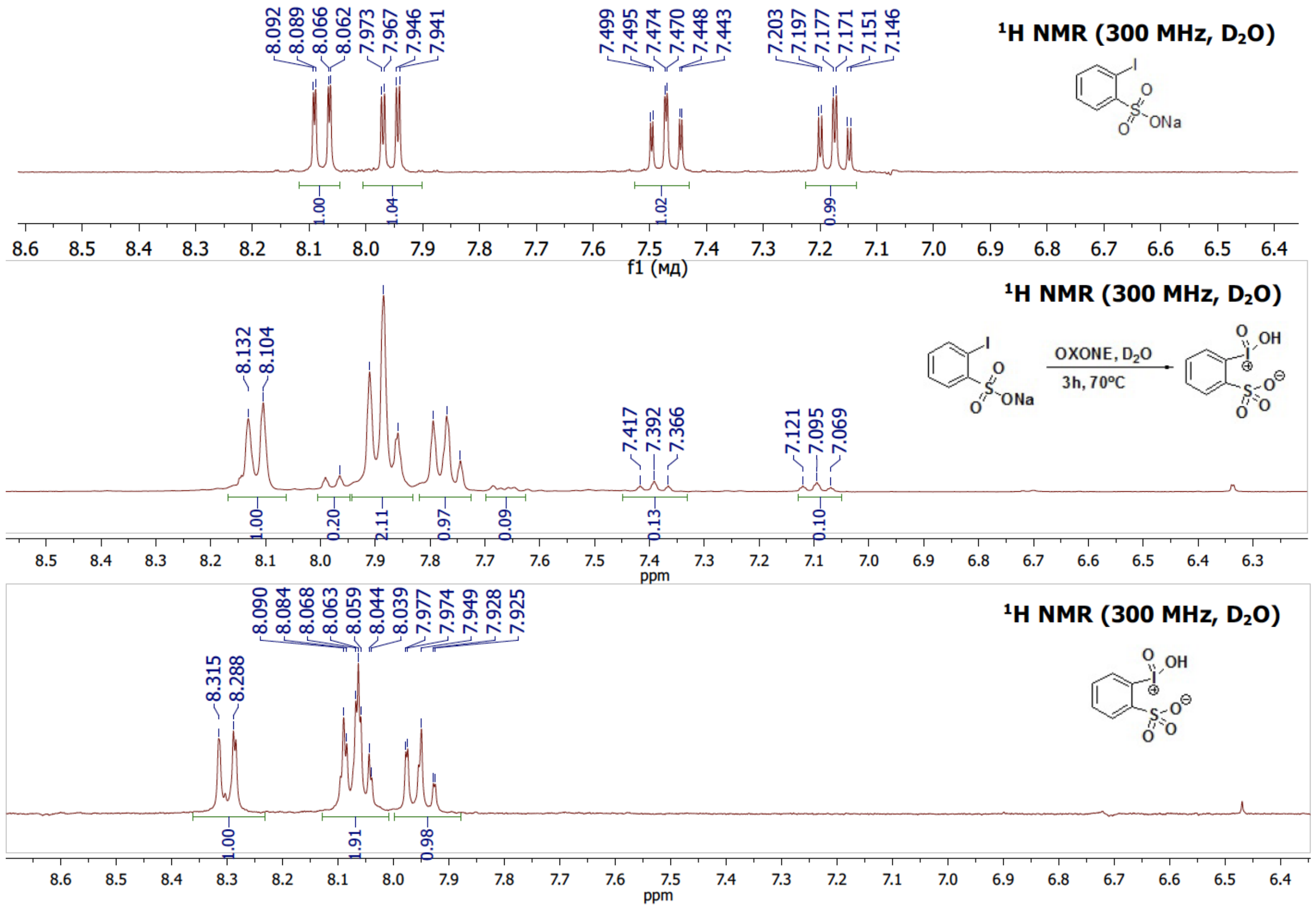

Figure S1: Dynamics of oxidation of sodium 2-iodobenzenesulfonate with Oxone at 70 ° C. ^{1}H NMR spectra: upper - signals of the initial sodium 2-iodobenzenesulfonate, medium - after heating for 3 hours, lower - signals of the product of oxidation of sodium 2-iodobenzenesulfonate.

X-ray crystallography of product 6-K

The needle-shaped crystals of product **6-K** were used for single crystal X-ray diffraction data collection. Crystallographic studies were undertaken on single crystal mounted in paratone and studied

on an Agilent SuperNova Dual three-circle diffractometer using Cu Kα or Mo Kα radiation and a CCD detector. Measurements were made at temperatures maintained using an Oxford cryostream. Data were collected and integrated and data corrected for absorption using a numerical absorption correction based on gaussian integration over a multifaceted crystal model within CrysAlisPro.[3]

Product **6-K** crystallizes in the *P*-1 space group with lattice parameters listed in Table S1. The unit cell contains two tetramers, two water molecules and six potassium cations. The structure has been solved by intrinsic phasing method with SHELXT[4] and refined by full-matrix least squares against F^2 using Crystals for Windows.[5] Selected details of structure refinement along with important statistics are given in Table S1. The data can be obtained from The Cambridge Crystallographic Data Centre under the reference number CCDC 1839998.

Table S1: Crystallographic details for tetrameric compound **6-K**.

Crystal data:	
chemical formula	$C_{24}H_{19}I_4K_3O_{21}S_4$
M_r	1396.58
crystal system, space group	triclinic, *P*-1
temperature (K)	296
a, *b*, *c* (Å)	12.1580(5), 14.0099(6), 14.3011(5)
α, β, γ (°)	61.355(4), 65.376(4), 65.479(4)
V (Å^3)	1871.29(7)
radiation type	Mo Kα
m (mm^{-1})	3.972

crystal size (mm)	0.196 × 0.104 × 0.086
Data collection	
diffractometer	Agilent SuperNova Dual three-circle diffractometer
absorption correction	gaussian integration over a multifaceted crystal model within CrysAlisPro
T_{min}, T_{max}	0.53, 0.71
no. of measured, independent and observed [$I > 2s(I)$] reflections	31392, 12160, 6397
R_{int}	0.065
θ_{max} (°)	29.631
Refinement:	
$R[F^2 > 2s(F^2)]$, $wR(F^2)$, S	0.0303, 0.0696, 0.8916
no. of reflections	9020
no. of parameters	514
no. of restraints	3
H-atom treatment	H-atom parameters constrained
Dρ_{max}, Dρ_{min} (e Å^{-3})	2.12, -1.97
absolute structure	NA

Oxidation of sodium 2-iodobenzenesulfonate 5 by sodium periodate.

Sodium 2-iodobenzenesulfonate (**5**, 100 mg, 0.33 mmol) was dissolved in 0.5 mL of distilled water, then, heated to 60 °C. $NaIO_4$ (137 mg, 0.82 mmol) was dissolved in 1 mL of distilled water, and the resulting solution was added to the solution of sodium 2-iodobenzenesulfonate (**5**) by several portions in 15 min. The reaction mixture was stirred for 3 h at 60 °C and then cooled to room temperature. Then, 10% aqueous solution of $AgNO_3$ was added to the reaction mixture and a white precipitate was formed. The precipitate of silver salts was filtered off, and the mother liquor was concentrated using blowing air to about half of initial volume. The concentrated aqueous solution was left for several days resulting in the formation of microcrystalline precipitate of IBS **6**-Na. Product **6**-Na was obtained as an off-white solid; yield 68 mg (61%); mp 194-196 °C. ^{1}H NMR (400 MHz, D_2O) δ, ppm: 8.24 (d, *J* = 8 Hz, 1H), 8.00-7.97 (m, 2H), 7.87 (t, *J* = 8 Hz, 1H). ^{13}C NMR (100 MHz, D_2O) δ, ppm: 142.8, 139.3, 134.1, 133.4, 128.2, 122.2.

Oxidation of sodium 2-iodobenzenesulfonate **5** with periodic acid under similar conditions afforded IBS **6**-Na in a mixture with 2-iodosylbenzenesulfonic acid **4** (see Figure 3S).

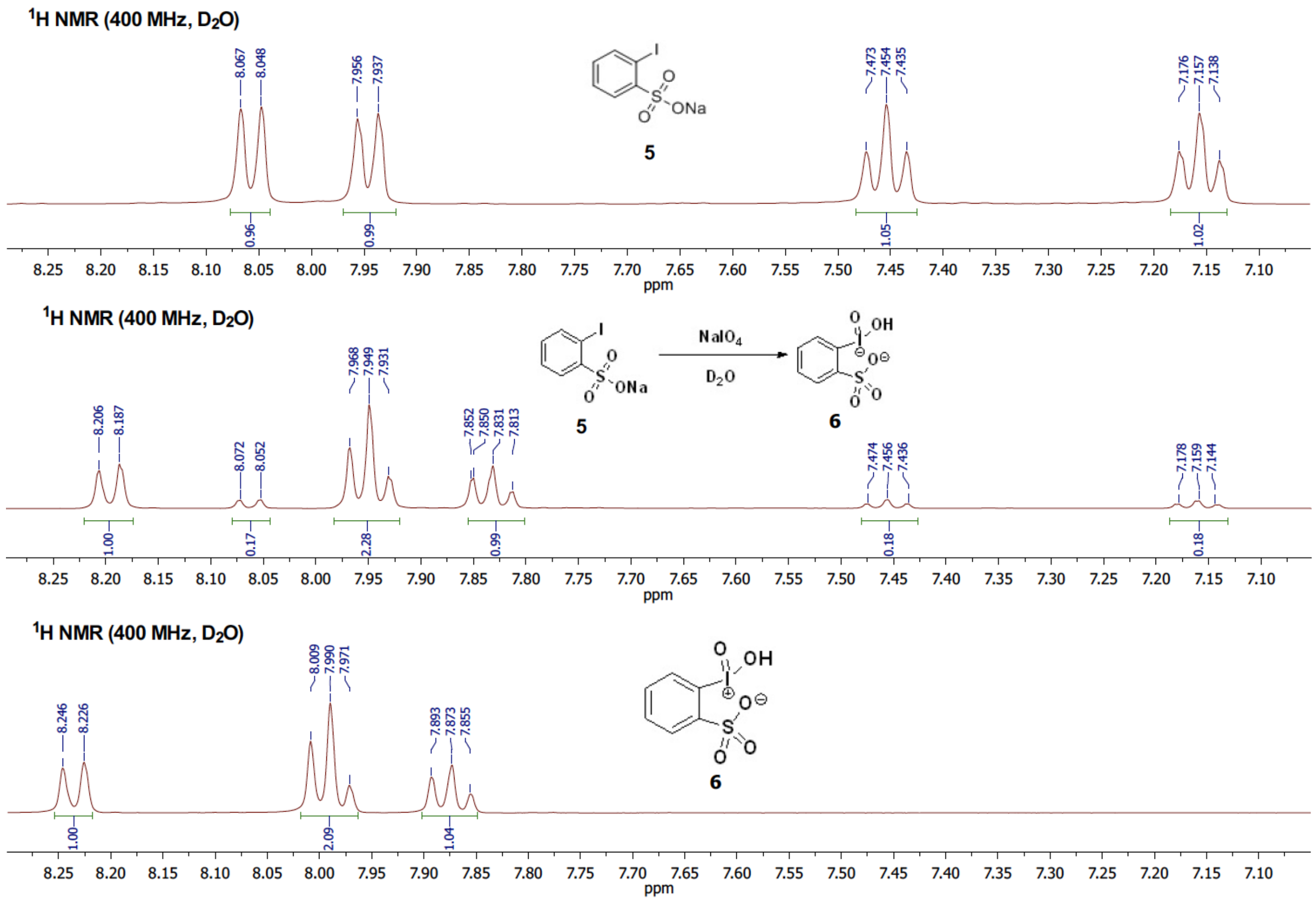

Figure S2: Dynamics of oxidation of sodium 2-iodobenzenesulfonate with sodium periodate at 60 °C. ^{1}H NMR spectra: upper - signals of the initial sodium 2-iodobenzenesulfonate, medium - after heating for 5 hours, lower - signals of the product of oxidation of sodium 2-iodobenzenesulfonate.

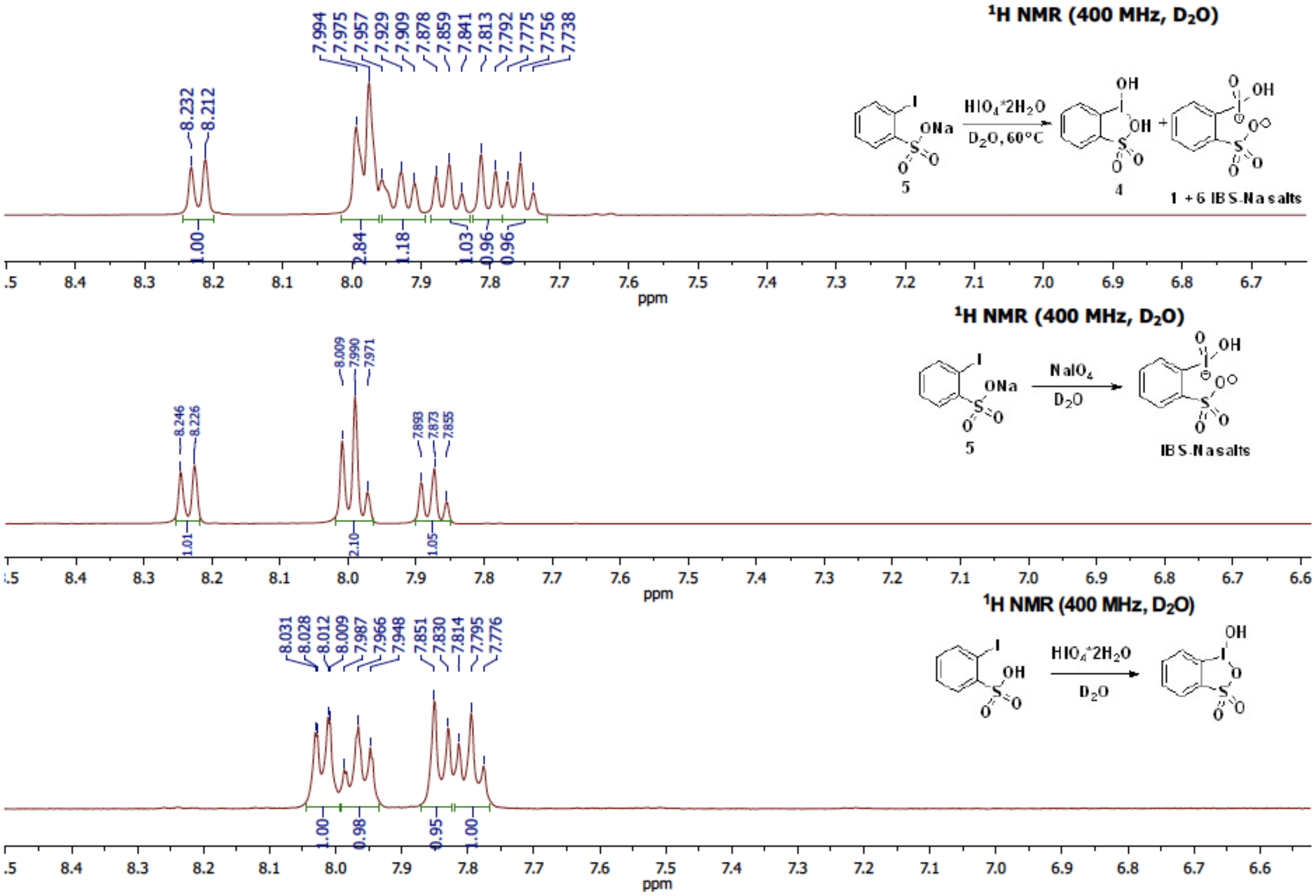

Figure S3: Oxidation of sodium 2-iodobenzenesulfonate with periodic acid at 60 °C in comparison with $NaIO_4$ under similar conditions.

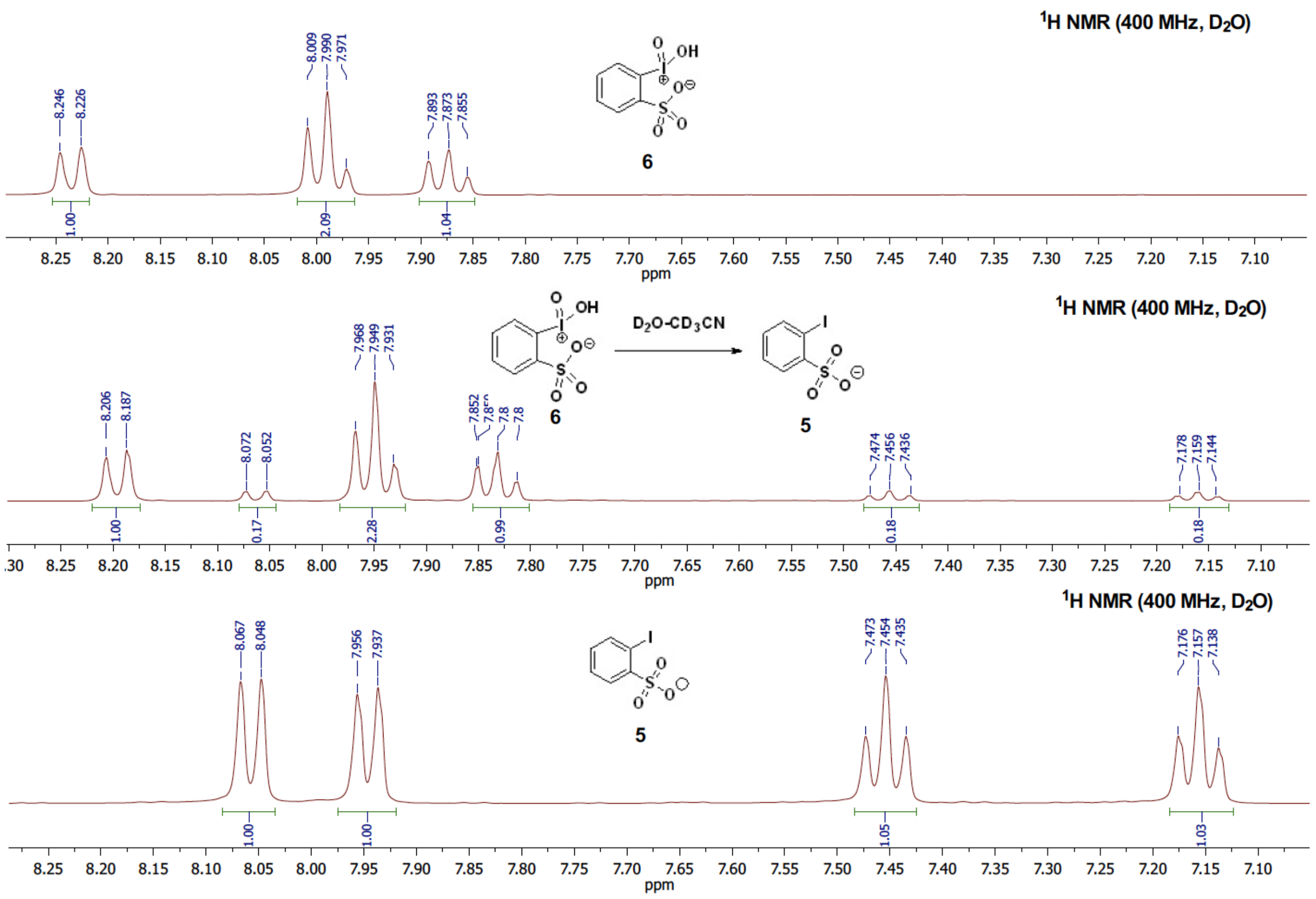

Figure S4: Dynamics of reduction of 2-iodoxybenzenesulfonic acid (IBS) by MeCN-d_3 at room temperature. ^{1}H NMR spectra: upper - signals of 2-iodoxybenzenesulfonic acid (IBS) in D_2O, medium – after addition of CD_3CN to aqueous solution of IBS, lower – signals of the product of reduction of IBS, that corresponds to sodium 2-iodobenzenesulfonate.

Oxidation of 2-iodobenzenesulfonic acid (7) by periodic acid.[6]

Periodic acid (1220 mg, 5.35 mmol) in 2 mL of distilled water was added to 2-iodobenzenesulfonic acid (500 mg, 1.76 mmol) in 1 mL of distilled water by one portion. The reaction mixture was heated to 60 °C and was stirred for about 5 hours. After that, the reaction mixture was cooled to room temperature, and a precipitate was formed. The white precipitate of 2-iodosylbenzenesulfonic acid (**4**) was filtered, washed with cold distilled water and dried in vacuo. Product **4** was obtained as a white solid; yield 485 mg (87%). Mp 125-130 °C. ^{1}H NMR (400 MHz, D_2O) δ, ppm: 7.99-7.91 (m, 2H),

7.82-7.74 (m, 2H). ^{13}C NMR (100 MHz, D_2O) δ, ppm: 137.8, 135.0, 131.9, 128.7, 125.8, 112.1.

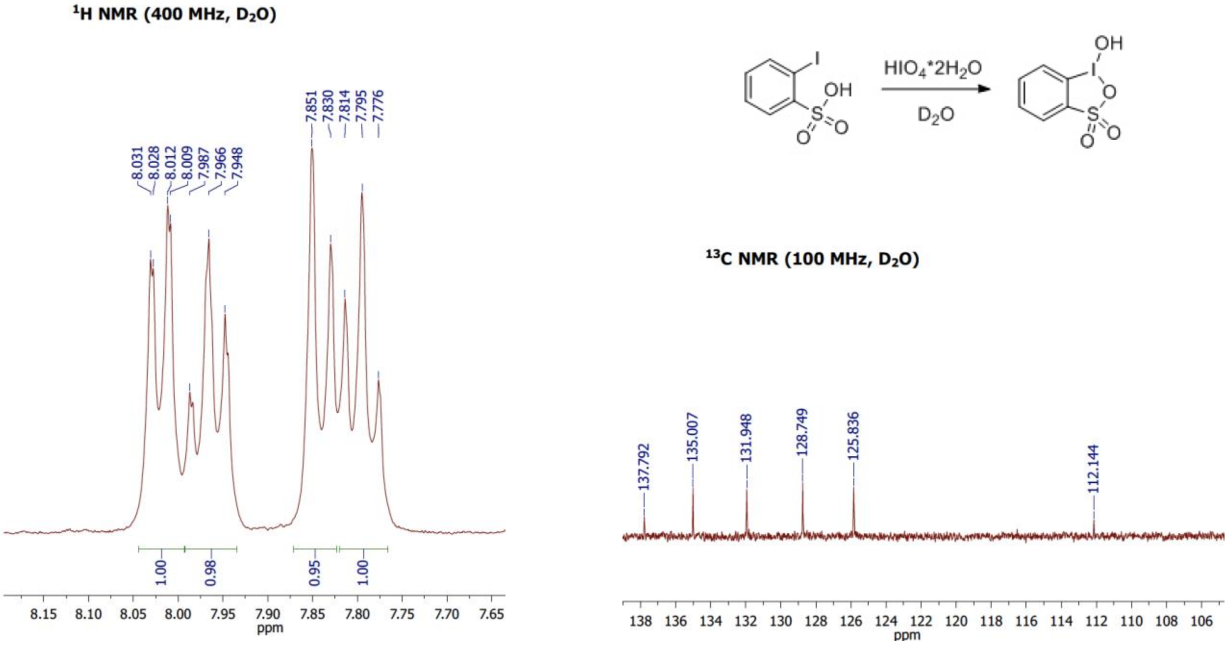

Figure S5: Oxidation of 2-iodobenzenesulfonic acid with periodic acid at 60 ºC for 5 hours.

References

1. Uyanik, M.; Ishihara, K.; Zhang, C.; Cui, L.-Q. 2-Iodobenzenesulfonic acid. *e-EROS Encyclopedia of Reagents for Organic Synthesis* 2012, 1-6.

2. Uyanik, M.; Akakura, M.; Ishihara, K. *J. Am. Chem. Soc.* **2009**, *131*, 251-262 [pp S50 and S51 of the Supporting Information

3. CrysAlisPro, Agilent Technologies, Version 1.171.37.33 (release 27-03-2014 CrysAlis171.NET).

4. Sheldrick, G. M. *Acta Crystallogr., Sect. A: Found. Adv.* **2015**, *71*, 3-8.

5. Betteridge, P. W.; Carruthers, J. R.; Cooper, R. I.; Prout, K.; Watkin, D. J. *J. Appl. Cryst.* **2003**, *36*, 1487.

6. Koser, G. F.; Sun, G.; Porter, C. W.; Youngs, W. J. *J. Org. Chem.* **1993**, *58*, 7310-7312.

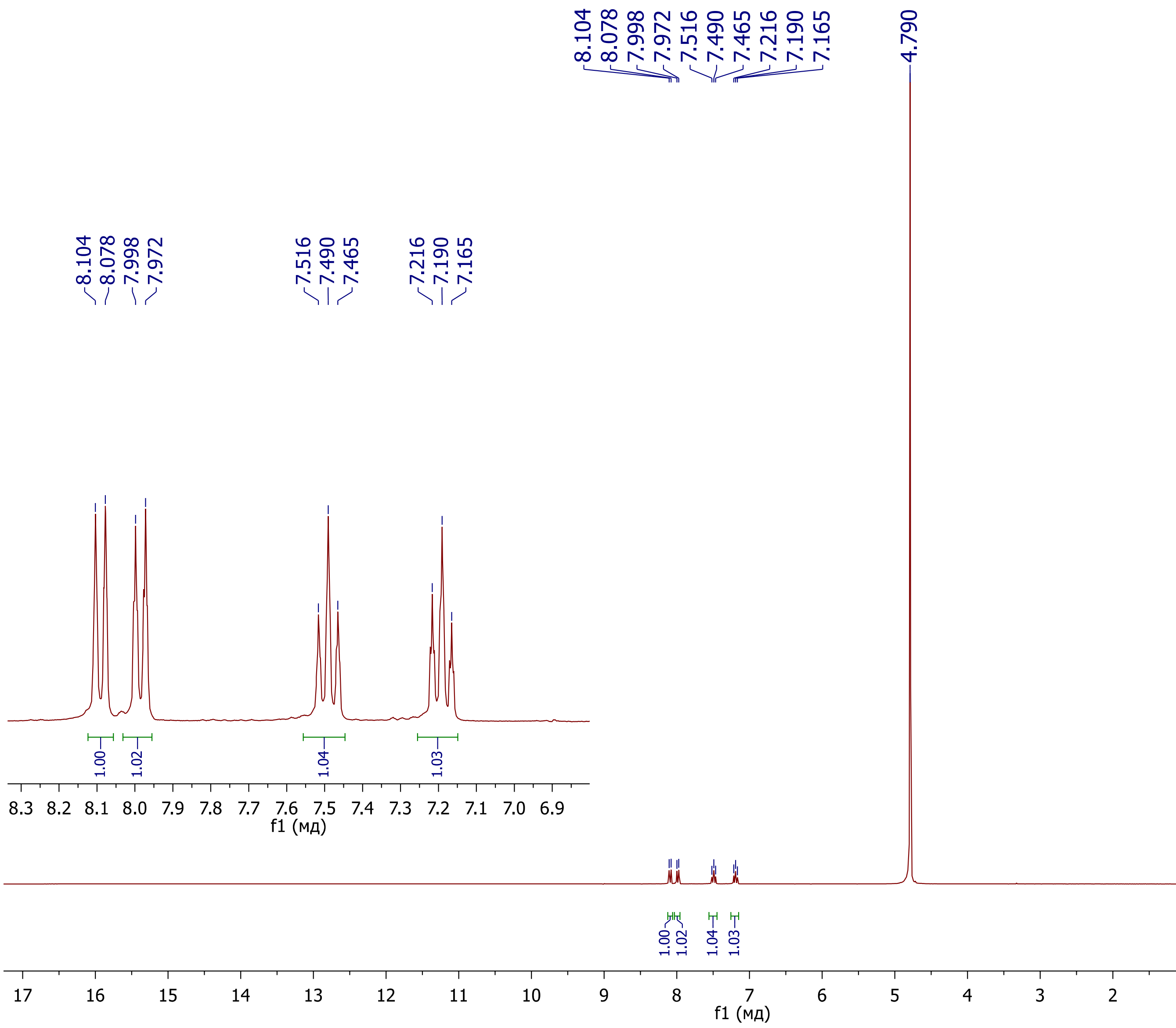

^{1}H NMR (300 MHz, D_2O)

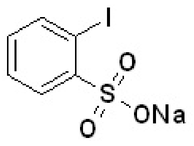

5

^{13}C NMR (100 MHz, D_2O)

5

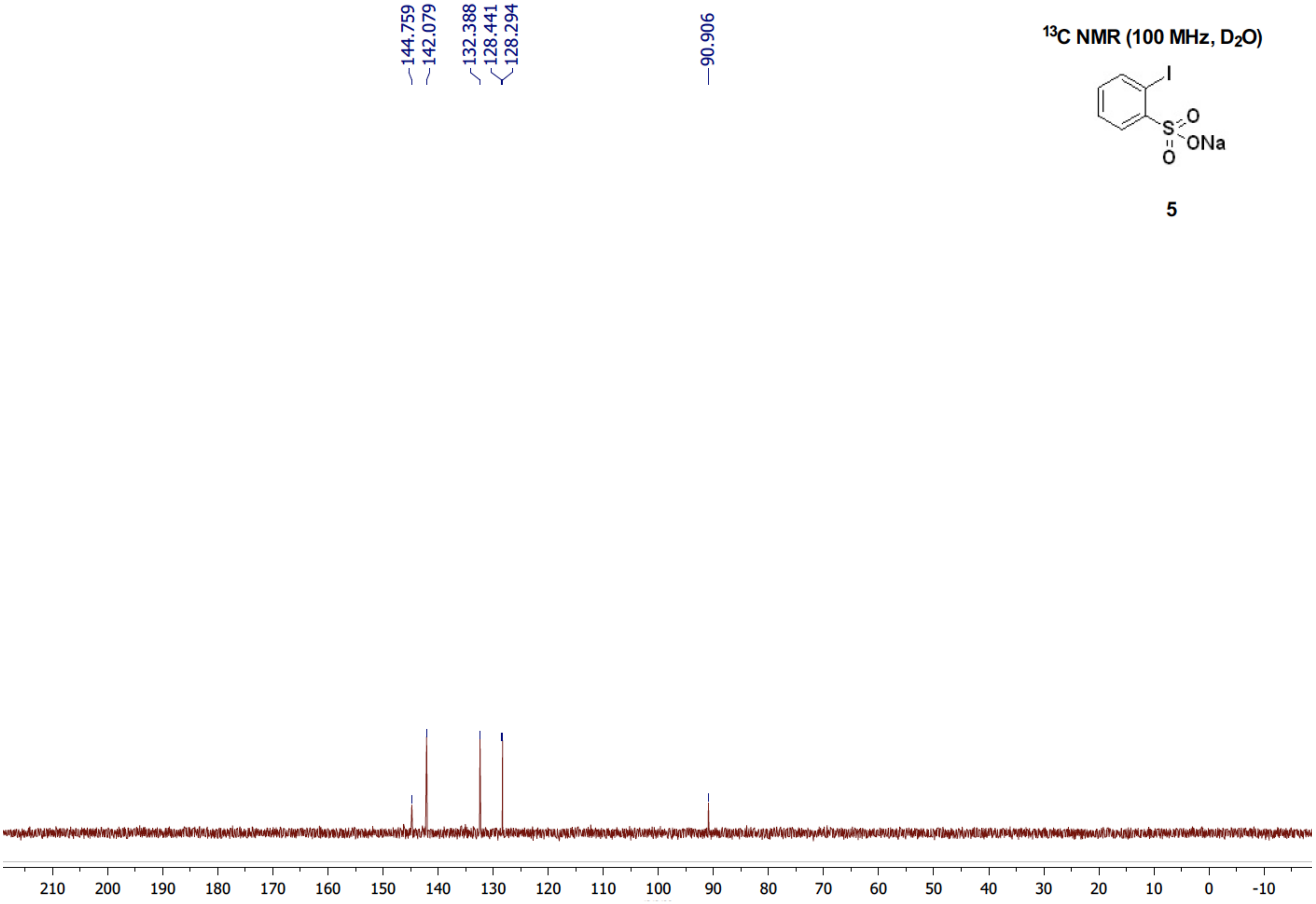

^1H NMR (400 MHz, D_2O)

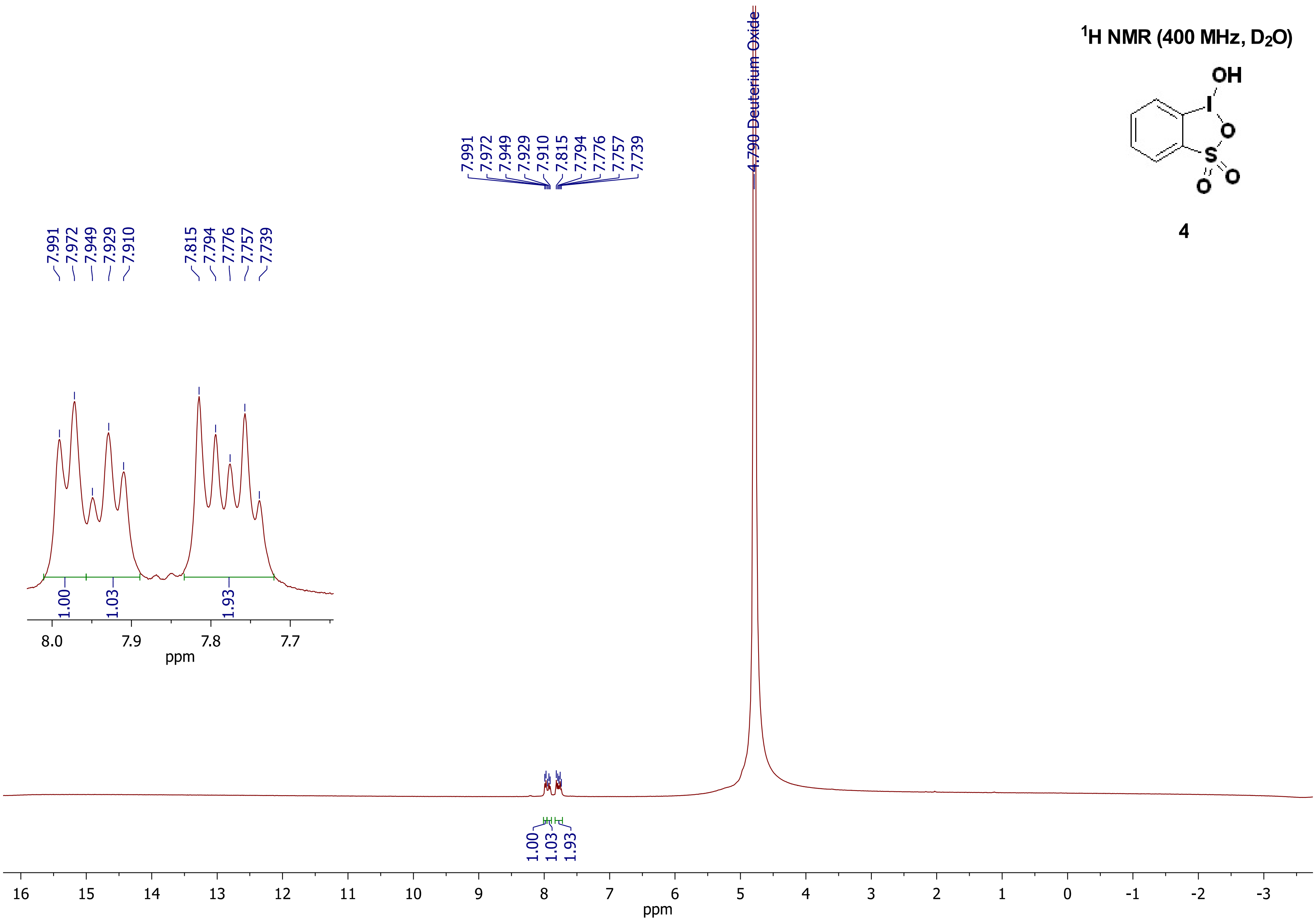

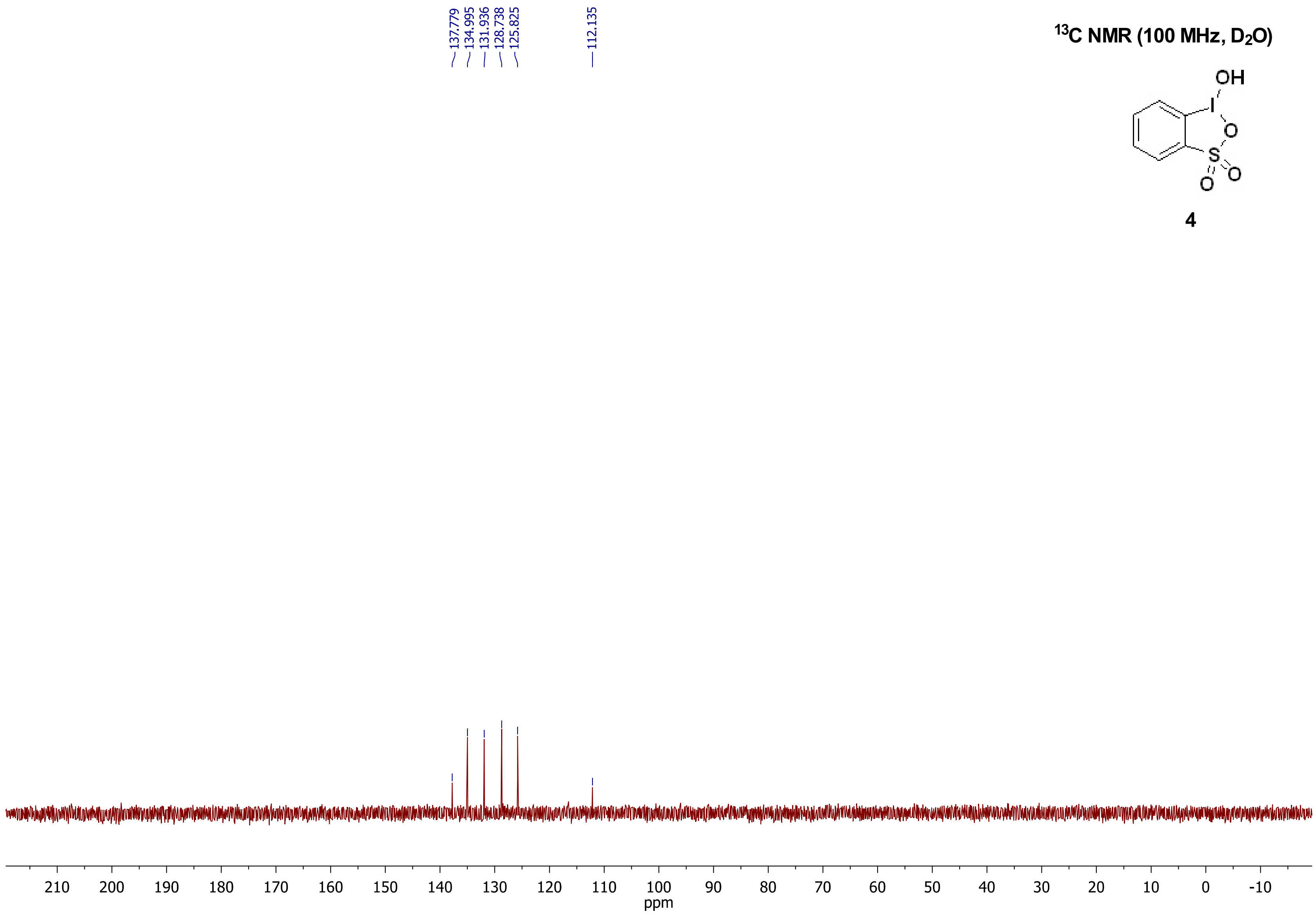

137.779
134.995
131.936
128.738
125.825
112.135
^{13}C NMR (100 MHz, D_2O)
OH
I
O
S
O
O
4
210 200 190 180 170 160 150 140 130 120 110 100 90 80 70 60 50 40 30 20 10 0 -10
ppm

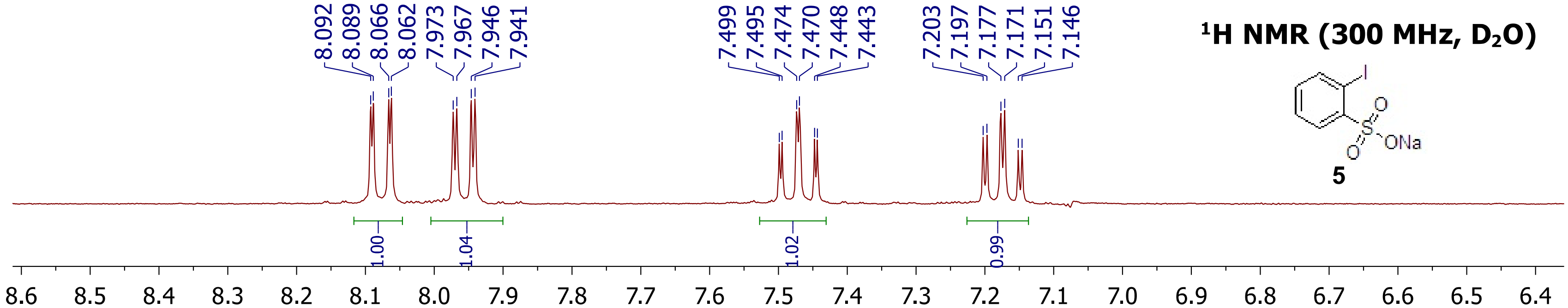
8.092 8.089 8.066 8.062 7.973 7.967 7.946 7.941
7.499 7.495 7.474 7.470 7.448 7.443
7.203 7.197 7.177 7.171 7.151 7.146
¹H NMR (300 MHz, D₂O)
I
O
S
O
ONa
5
1.00
1.04
1.02
0.99
8.6 8.5 8.4 8.3 8.2 8.1 8.0 7.9 7.8 7.7 7.6 7.5 7.4 7.3 7.2 7.1 7.0 6.9 6.8 6.7 6.6 6.5 6.4
f1 (мд)

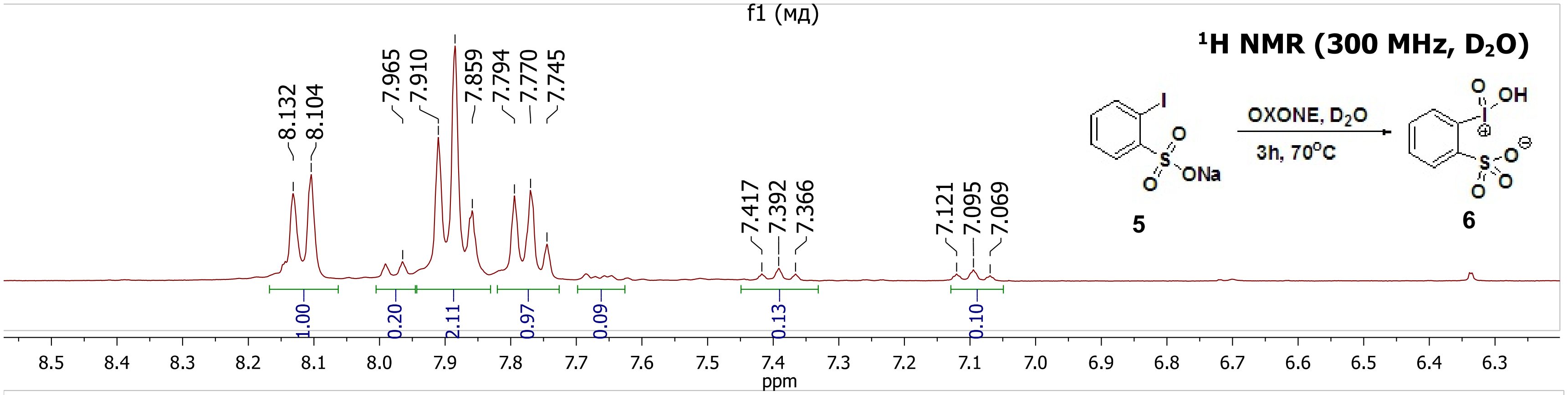
¹H NMR (300 MHz, D₂O)
8.132 8.104
7.965 7.910 7.859 7.794 7.770 7.745
7.417 7.392 7.366
7.121 7.095 7.069
I
O
S
O
ONa
5
OXONE, D₂O
3h, 70°C
O
OH
I
S
O
O
O
6
1.00
0.20
2.11
0.97
0.09
0.13
0.10
8.5 8.4 8.3 8.2 8.1 8.0 7.9 7.8 7.7 7.6 7.5 7.4 7.3 7.2 7.1 7.0 6.9 6.8 6.7 6.6 6.5 6.4 6.3
ppm

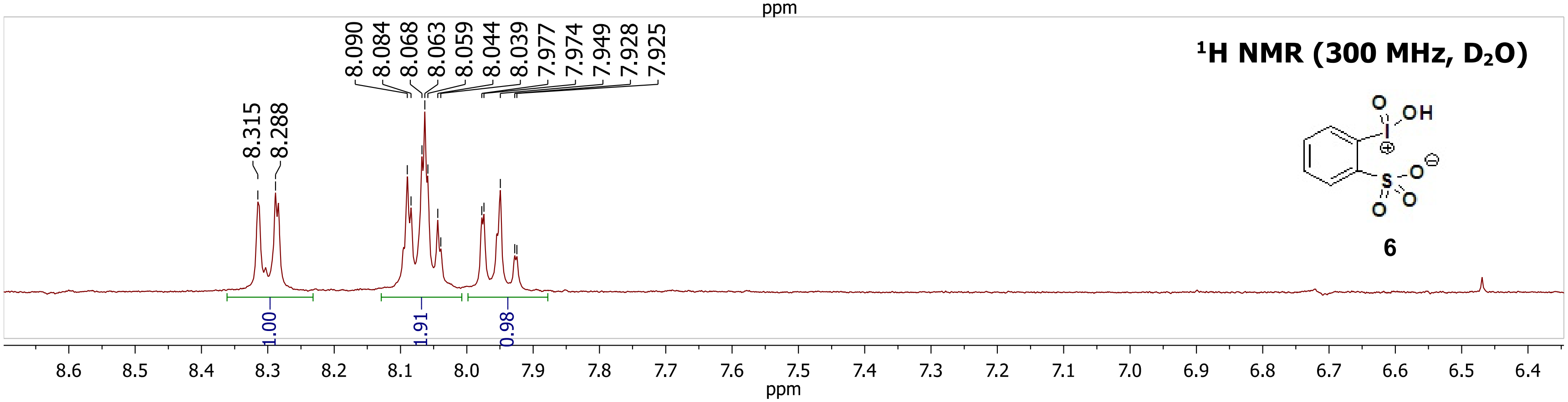
8.090 8.084 8.068 8.063 8.059 8.044 8.039 7.977 7.974 7.949 7.928 7.925
8.315 8.288
¹H NMR (300 MHz, D₂O)
O
OH
I
S
O
O
O
6
1.00
1.91
0.98
8.6 8.5 8.4 8.3 8.2 8.1 8.0 7.9 7.8 7.7 7.6 7.5 7.4 7.3 7.2 7.1 7.0 6.9 6.8 6.7 6.6 6.5 6.4
ppm

^1H NMR (300 MHz, D_2O)

6

OXONE as oxidant

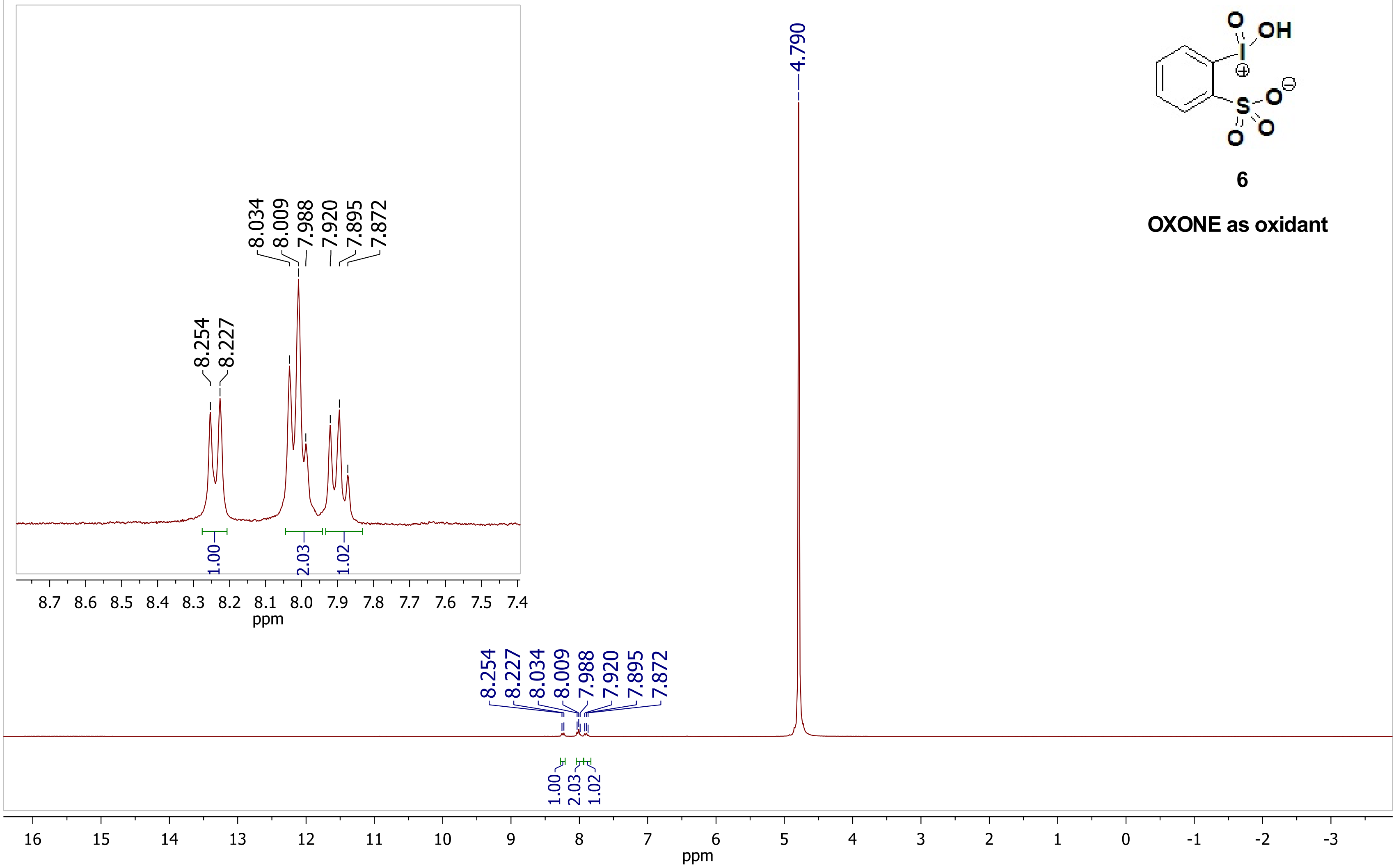

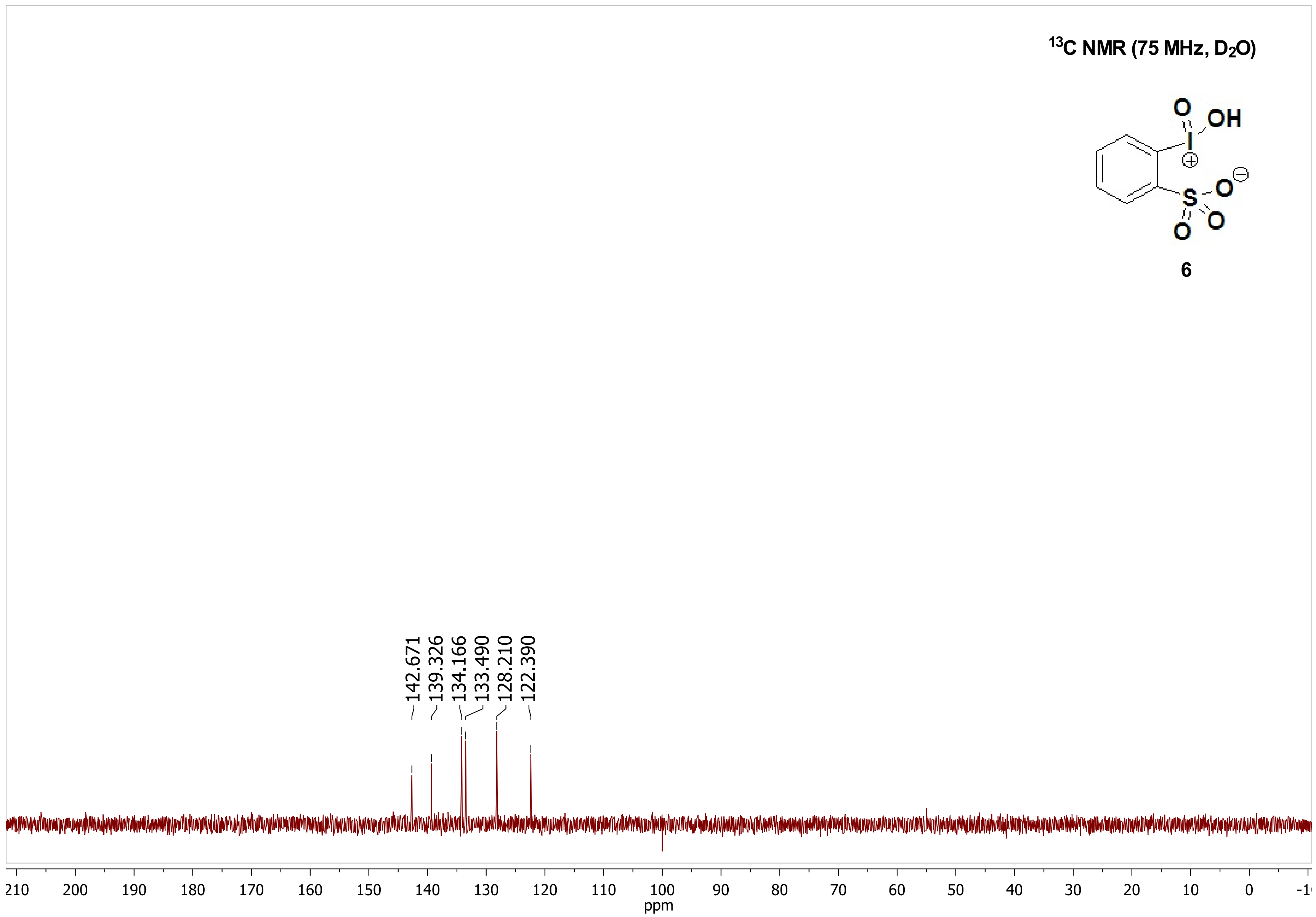

^{13}C NMR (75 MHz, D_2O)
OH
O
S
6
142.671
139.326
134.166
133.490
128.210
122.390
210
200
190
180
170
160
150
140
130
120
110
100
90
80
70
60
50
40
30
20
10
0
ppm

^{1}H NMR (400 MHz, D_2O)

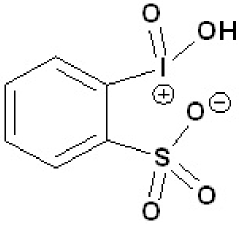

6

$NaIO_4$ as oxidante

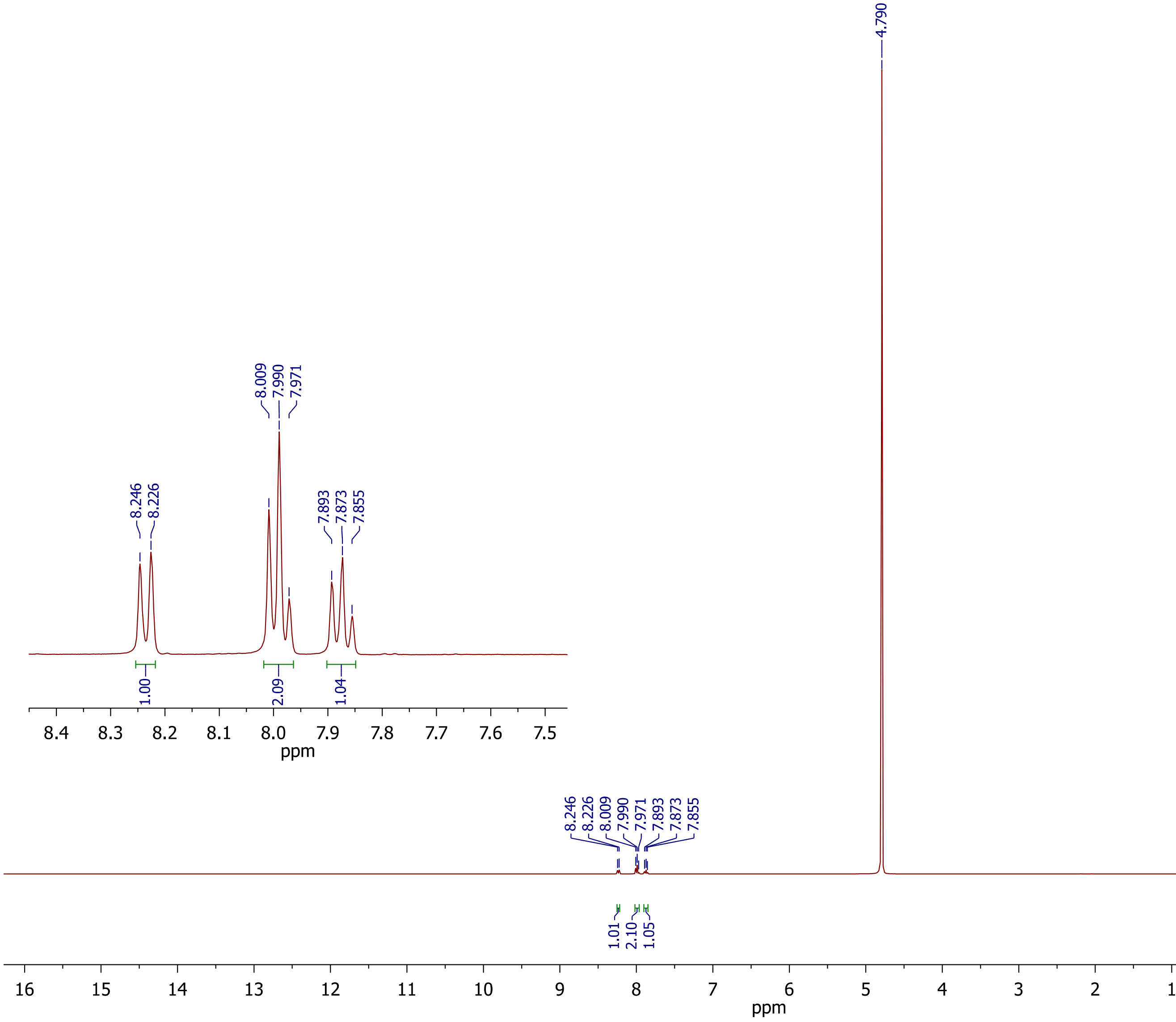

^{13}C NMR (100 MHz, D_2O)

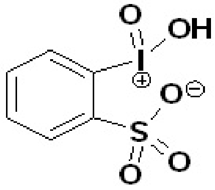

6

$NaIO_4$ as oxidante

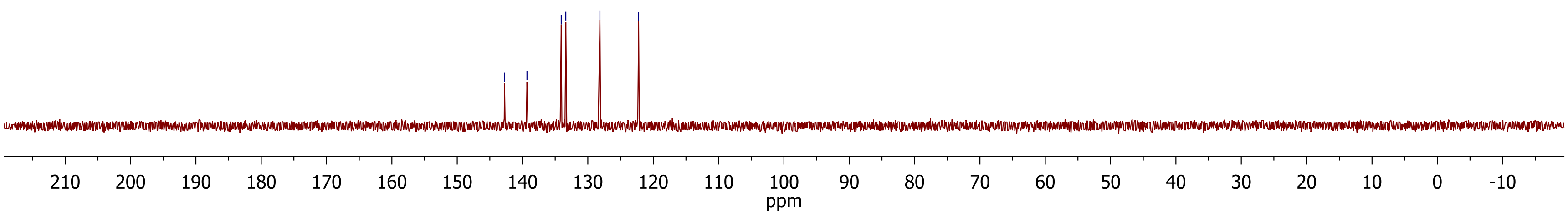

Supplement: File 1 — Experimental details and NMR spectra. [file Beilstein_J_Org_Chem-14-1854-s001.pdf]
